# Age-Associated Proteomic Changes in Human Spermatozoa

**DOI:** 10.3390/ijms26136099

**Published:** 2025-06-25

**Authors:** Mohd Amin Beg, Abrar Osama Ismail, Ayodele Alaiya, Firdous Ahmad Khan, Taha Abo-Almagd Abdel-Meguid Hamoda, Ishfaq Ahmad Sheikh, Priyanka Sharma, Omar Mohammed Baothman, Ali Hasan Alkhzaim, Zakia Shinwari, Rinad Fahad Abuzinadah, Arif Mohammed, Abdullah Mohammed Assiri, Adel Mohammad Abuzenadah, Erdogan Memili, Jean Magloire Feugang

**Affiliations:** 1 King Fahd Medical Research Center, King Abdulaziz University, Jeddah 21589, Saudi Arabia; abrar.ismail1992@gmail.com (A.O.I.); iasheikh@kau.edu.sa (I.A.S.); aabuzenadah@kau.edu.sa (A.M.A.); 2 Biomedical Sciences, Clinical Embryology and Reproductive Biology, College of Medicine, Alfaisal University, Riyadh 11533, Saudi Arabia; 3 Cell Therapy & Immunobiology Department, King Faisal Specialist Hospital and Research Center, Riyadh 11211, Saudi Arabia; aalaiya@kfshrc.edu.sa (A.A.); szakia@kfshrc.edu.sa (Z.S.); 4 Department of Large Animal Medicine and Surgery, School of Veterinary Medicine, St. George’s University, True Blue, Grenada; fkhan8@sgu.edu; 5 Department of Urology, Faculty of Medicine, King Abdulaziz University, Jeddah 21589, Saudi Arabia; tahaaboalmagd@yahoo.com (T.A.-A.A.-M.H.); ali.ali-14@hotmail.com (A.H.A.); 6 Center of Innovation in Personalized Medicine, King Abdulaziz University, Jeddah 21589, Saudi Arabia; priyanka82jnu@gmail.com (P.S.); rfabuzinadah@kau.edu.sa (R.F.A.); 7 Microbiology and Molecular Section, Al Salama Hospital, Jeddah 23611, Saudi Arabia; baothman30@hotmail.com; 8 Department of Biological Sciences, College of Science, University of Jeddah, Jeddah 23890, Saudi Arabia; aarifsls@yahoo.co.in; 9 Department of Comparative Medicine, King Faisal Specialist Hospital and Research Center, Riyadh 11211, Saudi Arabia; assiri@kfshrc.edu.sa; 10 King Faisal University, Hufof 36362, Saudi Arabia; 11 Cooperative Agricultural Research Center, College of Agriculture and Human Sciences, Prairie View A&M University, Prairie View, TX 77446, USA; ermemili@pvamu.edu; 12 Department of Animal and Dairy Sciences, Mississippi State University, Starkville, MS 39759, USA; j.feugang@msstate.edu

**Keywords:** human, aging, spermatozoa, proteomics, LC-MS/MS

## Abstract

Advancing age in men significantly contributes to declining sperm fertility. Information on age-related proteomic changes in spermatozoa is limited. This study involved normal fertile Arab men in three age groups: young adult (21–30 years; *n* = 6), late adult (31–40 years; *n* = 7), and advanced age (40–51 years; *n* = 5). Gradient-purified spermatozoa were analyzed using LC-MS/MS and proteomic data were processed using Progenesis QI (QIfp) v3.0 and UniProt/SwissProt. Significantly enriched annotations and clustering of proteins in the proteomic datasets were identified (2-fold change; *p* < 0.05). A total of 588 proteins were identified, with 93% shared across the three groups. Unique proteins were MYLK4 for the young adult group, PRSS57 for the late adult group, and HMGB4, KRT4, LPGAT1, OXCT2, and MGRN1 for the advanced age group. Furthermore, 261 (44%) proteins were differentially expressed (*p* < 0.05) across the three groups. Functional enrichment analysis suggested an aging-related significant increase in pathways associated with neurodegenerative diseases and protein folding, alongside decreases in glycolysis/gluconeogenesis, flagellated sperm motility, acetylation, phosphoprotein modifications, oxidation processes, and Ubl conjugation. Cluster analysis highlighted significantly upregulated proteins in young adults (e.g., H2BC1, LAP3, SQLE, LTF, PDIA4, DYNLT2) and late adults (e.g., ATP5F1B, ODF2, TUBA3C, ENO1, SPO11, TEX45, TEKT3), whereas most proteins in the advanced age group exhibited downregulation (e.g., SPESP1, RAB10, SEPTIN4, RAB15, PTPN7, USP5, ANXA1, PRDX1). In conclusion, this study revealed aging-associated proteomic changes in spermatozoa that impact critical processes, including spermatogenesis, motility, metabolism, and fertilization, potentially contributing to fertility decline. These changes provide a molecular framework for developing therapies to preserve sperm proteostasis and enhance fertility in older men.

## 1. Introduction

Fertility in men is significantly affected by age [1,2,3,4,5,6]. As sperm production continues, age brings structural and functional changes that negatively impact fertility [7,8]. Aging affects testicular histomorphology, semen characteristics, oxidative stress, genetics, and the endocrine system [1,5,9]. Key challenges include altered testosterone levels [10], declines in sperm count and quality [6,8], increased DNA fragmentation [1,5,11], and sperm cell membrane integrity [7]. Older men also face higher risks of diabetes and obesity, further impacting fertility and increasing early miscarriage risks [12,13,14]. Additionally, age is linked to chromosomal and epigenetic abnormalities that can affect embryonic development and long-term offspring health [15,16].

Modern lifestyles and rapid industrialization have led to more individuals delaying parenthood for various reasons, such as financial concerns, career pursuit, higher education opportunities, improved contraceptive methods, divorce and remarriage, high childcare costs, and a desire to enjoy life [17,18]. An analysis of natality data from the National Center for Health Statistics (NCHS) of the United States revealed that from 1972 to 2015, the average paternal age increased from 27.4 to 31.9 years [19]. The percentage of fathers aged 40 and older rose from 4.1% to 8.9%, and those aged 50 and above grew from 0.5% to 0.9%. Additionally, the birth rate among men aged 35 to 49 in 2015 was 1.6 times higher than in 1980 [20]. Similar trends are anticipated in low- and middle-income countries as they experience demographic shifts toward an older population [21]. In Saudi Arabia, trends driven by industrialization and social changes are leading to more individuals from both genders pursuing higher education and entering the workforce [22,23,24,25]. Although specific data on paternal age distribution is lacking, projections indicate that the median age in the Kingdom will rise from 29 years in 2020 to 40 years by 2050, with men aged 35 to 49 making up the largest group of the male reproductive age population [26,27]. Globally, this demographic shift underscores the growing significance of aging in public health, linked to genetic risks, reproductive challenges, and potential health effects on future generations [17,28,29]. Research using U.S. NCHS data estimated a 4–13.2% rise in instances of various diseases, such as leukemia, schizophrenia, and autism spectrum disorder, and breast cancer among the 2015 birth cohort compared to that of 1972 [30].

The effects of advanced paternal age on fertility have historically received limited attention, leading to an incomplete understanding of this area. Studies show age-related impacts in men, including histological changes and a decrease in Sertoli, Leydig, and germ cells [31,32]. Men may experience lower androgen levels and erectile dysfunction [1,33]. Noticeable adverse changes begin to occur in semen parameters in men around 40 years of age [1,32]. This includes deterioration in semen quality, characterized by abnormalities in ejaculate volume, morphology, concentration, total count, motility, and viscosity [7,34,35,36,37]. Additionally, increased delay in pregnancy onset and declining probability for natural conception, along with lower pregnancy and birth rates by in vitro fertilization (IVF), have been reported [1,2,32]. Aging is also linked to increased infection, oxidative stress, obesity, sperm DNA fragmentation, longer conception times, higher miscarriage rates, and negative effects on the outcomes of assisted reproductive technologies [1,35,38]. For couples with undiagnosed infertility, the impact of paternal age can be particularly significant, as precious time is lost before recognizing the implications of age. A study of 21,960 injected oocytes from an intracytoplasmic sperm injection (ICSI) program found that each additional year of paternal age reduced the odds of live birth by 1% for women aged 37, 1.6% for those aged 38, 2.4% for those aged 39, and 5% for those aged 42 [39].

Aging significantly affects male fertility, highlighting the need to study age-related molecular and functional genomics, especially sperm proteomics. Several comprehensive reviews of the proteomics of spermatozoa highlight its significance in sperm biology, clinical diagnostics, and infertility research [40,41,42,43]. Proteomics has significantly advanced our understanding of the role of proteins in spermatogenesis, as well as key sperm functions such as motility and fertilizing ability. Moreover, proteomics provides valuable insights into post-translational modifications (PTMs) such as acetylation, methylation, and phosphorylation within the sperm proteome, along with the molecular pathways, cellular mechanisms, and protein–protein interactions essential for normal reproductive processes and the underlying causes of infertility [41,42,43]. Proteomic profiling has revealed significant differences between the sperm of fertile men and those with various infertility conditions. A study comparing the sperm proteomes of asthenozoospermic and fertile men identified 17 differentially expressed proteins (DEPs), including cytoskeletal actin-B, annexin-A5, histone H2A, and heat shock protein HSPA2 [44]. Similarly, another study on asthenozoospermic samples identified altered expression in 25 sperm proteins, many of which are involved in energy metabolism [45]. Men with asthenozoospermia or oligoasthenoteratozoospermia also exhibited distinct PTMs in their sperm nuclear protamines [46]. Both protamine 1 and protamine 2 showed altered phosphorylation and methylation patterns, with overall protamine 1 phosphorylation significantly higher in infertile men than in fertile men [46]. Spermatozoa from semen samples with high oxidative stress exhibited differential expression in 15 proteins related to energy and carbohydrate metabolism, protein modifications, and oxidative stress regulation [47]. In another study, comparisons between unexplained infertile men and fertile men revealed 162 sperm DEPs [48]. Upregulated DEPs were associated with the enrichment of pathways related to free radical scavenging. In contrast, downregulated DEPs were linked to reproductive system development and function, as well as to the ubiquitination pathway [48]. Furthermore, even among fertile men, comparing high-motility sperm to low-motility sperm within the same ejaculate revealed 21 DEPs [49]. Many of these DEPs function as molecular chaperones; notably, HSPA1L and HSPA9 were significantly less abundant in the low-motility sperm fraction. Oligozoospermic men also displayed distinct sperm proteomic profiles compared to fertile men, with DEPs primarily involved in flagellar assembly and sperm motility, fertilization, and spermatogenesis [50]. A set of 18 highly differential proteins, including C2orf16, CYLC1, SPATA31E1, SPATA31D1, SPATA48, EFHB (CFAP21), and FAM161A, was identified as potential diagnostic markers of oligospermic infertility [50]. Sperm proteins are vital for early embryonic development and can be biomarkers for successful in vitro fertilization [51].

Despite the well-documented effects of aging on male fertility, research on age-related changes in the human sperm proteome is limited, with only two studies having been conducted [52,53]. The first study compared the sperm proteomes of men aged 28–32 and 68–72 years. Still, it only analyzed 22 protein spots and focused on the protein prostate and testis expressed 1 (PATE1), a protein essential for sperm–egg penetration and motility [52]. The second study examined sperm from men aged 26–30 and 45–54 years, finding 80 proteins with significant alterations—64 upregulated and 16 downregulated in the older group—linked to pathways related to Parkinson’s disease and cholesterol metabolism. However, since the participants were undergoing fertility treatment, the findings may have also been affected by related challenges [53]. Thus, more research is needed on men with normal fertility across defined age groups to accurately evaluate age-related proteomic changes in sperm.

This study examines how aging influences the proteomic landscape of human spermatozoa. Proteomic analysis was performed on sperm samples from three stratified age groups of normal, fertile men: young adults (21–30 years), late adults (31–40 years), and advanced age (41–51 years). The analysis revealed dynamic age-related alterations in human sperm proteins potentially impacting key sperm physiological functions such as motility, metabolism, and fertilization.

## 2. Results

### 2.1. Semen Analysis

Table 1 summarizes the mean data (±SD) collected from all three age groups of men (young adult, late adult, and advanced age). The data includes age, BMI, and abstinence period, along with corresponding semen parameters (ejaculate volume, sperm concentration, sperm motility, sperm progression, total sperms per ejaculate, total motile sperms per ejaculate, and sperm morphology). The mean age for each group differed significantly (*p* < 0.001) from the other two groups. The late adult group had the highest sperm concentration, significantly different (*p* < 0.02) from the young adult group, while the other group comparisons showed no significant differences. All other parameters showed no significant differences among the age groups (Table 1).

### 2.2. Protein Detection and Functional Analysis

**Figure 1:**
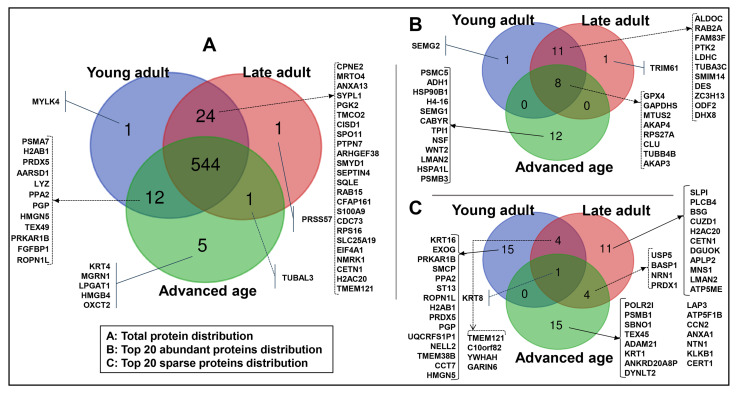
Venn diagram of total (**A**), top 20 abundant (**B**), and top 20 sparse (**C**) protein distribution of spermatozoa across the age groups. The age groups of men are young adult (21–30 years), late adult (31–40 years), and advanced age (41–51 years).

A total of 588 unique proteins were detected in the young adult (581), late adult (570), and advanced age (562) groups (Supplementary Data File S1). The Venn diagram shows the protein distribution across the age groups (Figure 1). The total proteomes revealed 544 (93%) proteins found across all groups (Figure 1A). In contrast, a few other proteins were detected in two or individual groups (specific proteins). These specific proteins were found in each group: the young adult group—myosin light chain kinase family member 4 (MYLK4); the late adult group—serine protease 57 (PRSS57); and the advanced age group—keratin_type II cytoskeletal 4 (KRT4), E3 ubiquitin-protein ligase (MGRN1), acyl-CoA:lysophosphatidylglycerol acyltransferase 1 (LPGAT1), high-mobility group protein B4 (HMGB4), and succinyl-CoA:3-ketoacid coenzyme A transferase 2, mitochondrial (OXCT2). In addition, 24 proteins were specific to only the young adult and late adult groups, 12 proteins only to the young adult and advanced age groups, and 1 protein only to the late adult and advanced age groups (Figure 1A). The top 20 expressed proteins in each group corresponded to 33 unique proteins, with about 24% (8/33) shared across the three groups (Figure 1B). In contrast, a total of 50 proteins were unique among the bottom 20 expressed proteins in each group, and only 1 of 50 proteins was shared between the three groups (Figure 1C). Further details of detected protein characteristics are provided in Supplementary Data File S1.

**Figure 2:**
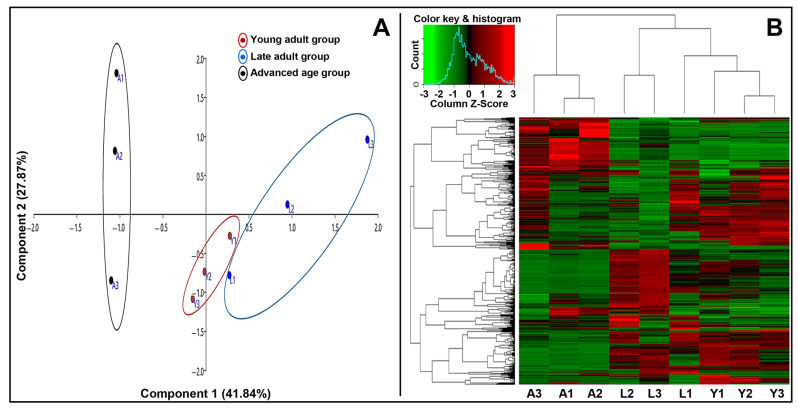
Principal component analysis (**A**) and hierarchical heat map (**B**) of sperm proteome during aging. The age groups are young adult (Y1–Y3; 21–30 years), late adult (L1–L3; 31–40 years), and advanced age (A1–A3; 41–51 years).

A total of 261 proteins (44%) were differentially expressed (*p* < 0.05) across the three age groups, including two proteins that ranked among the top 20 most abundant across all age groups (AKAP4 and GPX4), which were significantly downregulated in the advanced age group. Figure 2 shows the principal component analysis (PCA) depicting age groups without overlapping (Figure 2A), while the hierarchical heat map indicates an overview of the differential protein expression among the age groups (Figure 2B). Further details of differential proteins are provided in Supplementary Data File S1.

**Figure 3:**
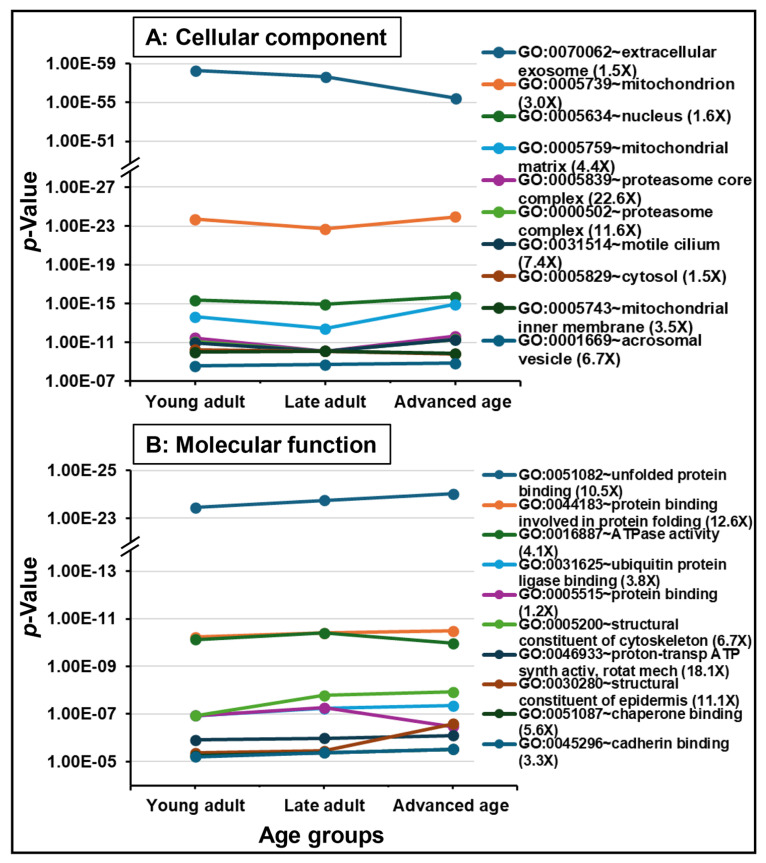
Functional enrichment analysis of the sperm proteome during aging. The top 10 significantly enriched GO terms (*p* < 0.05) in the Cellular component and Molecular function categories are shown. The fold enrichment of each GO term is indicated in parentheses. The age groups of men are young adult (21–30 years), late adult (31–40 years), and advanced age (41–51 years).

**Figure 4:**
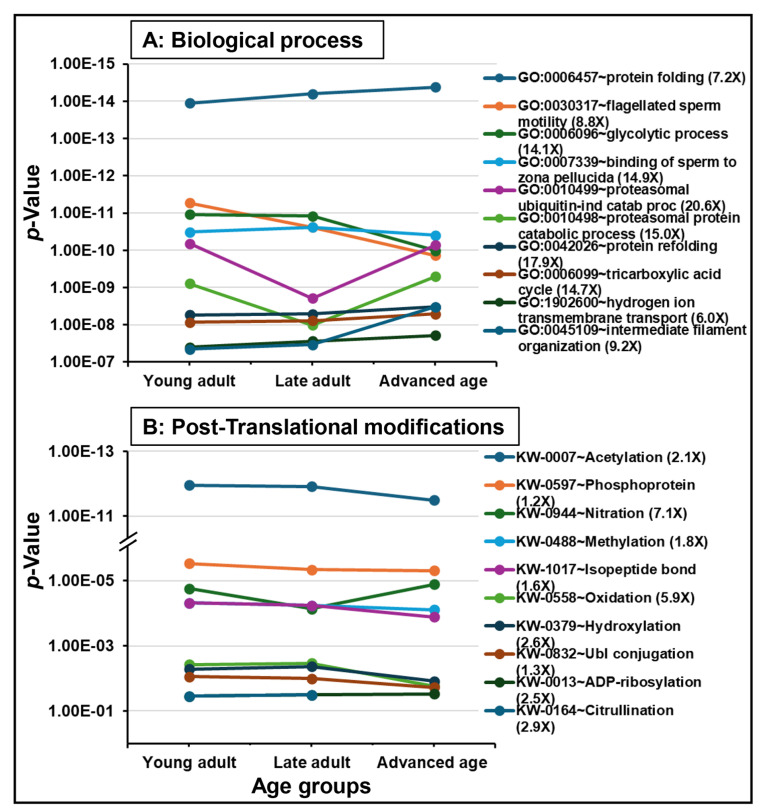
Functional enrichment analysis of the sperm proteome during aging. The top 10 significantly enriched GO terms (*p* < 0.05) in the Biological category and post-translational modification terms or PTMs are shown. The fold enrichment of each GO term is indicated in parentheses. The age groups of men are young adult (21–30 years), late adult (31–40 years), and advanced age (41–51 years).

**Figure 5:**
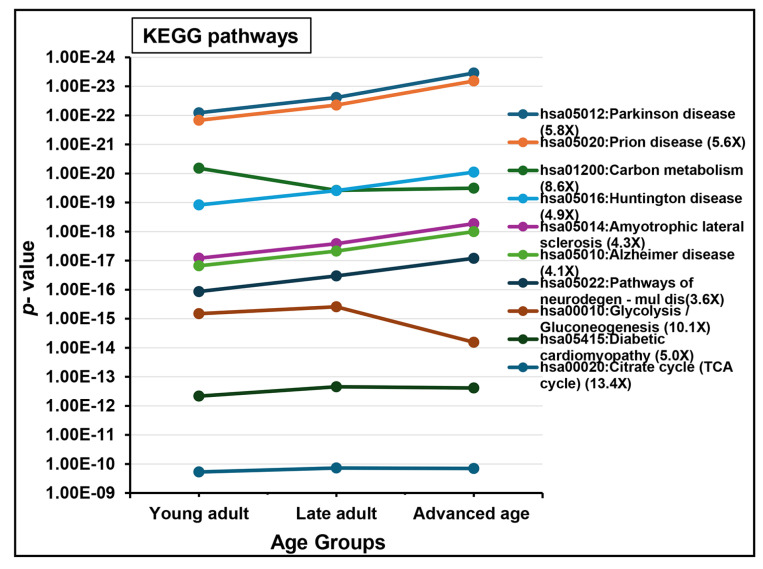
KEGG pathway analyses of sperm proteome during aging. The top 10 significantly (*p* < 0.05) enriched pathways are shown. The fold enrichment of each pathway term is indicated in parentheses. The age groups of men are young adult (21–30 years), late adult (31–40 years), and advanced age (41–51 years).

The functional analysis of all datasets revealed significant enrichments in biological functions. Figure 3 and Figure 4 represent the dynamics of significantly enriched biological functions in each ontology category and pathways during aging. Datasets were enriched with proteins associated with gene ontology (GO) terms belonging to cellular component (i.e., “extracellular exosome”, “mitochondrion”, “nucleus”; Figure 3A), molecular function (i.e., “unfolded protein binding”, “protein binding involved in protein folding”, “ATPase activity”; Figure 3B), and biological process (i.e., “protein folding”, “flagellated sperm motility”, “glycolytic process”; Figure 4A). Additionally, “acetylation” and “phosphoprotein” were found to be the most significantly enriched PTMs during aging (Figure 4B). Seven out of ten KEGG pathways were related to diseases such as “Parkinson disease”, “prion disease”, and “Huntington disease,” which were the highly significantly enriched KEGG pathways, continuously increasing during aging. The remaining KEGG pathways were “carbon metabolism”, “glycolysis/gluconeogenesis”, and “citrate cycle (TCA cycle)” (Figure 5).

**Figure 6:**
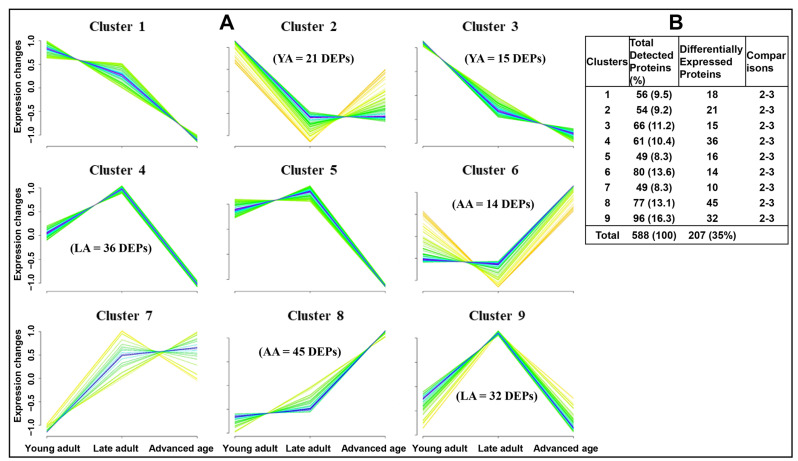
Clustering analysis (fuzzy C-means) of the sperm proteome during aging. Nine different protein expression profiles were detected (**A**), and substantial numbers of proteins were distributed across clusters (**B**). Each line in the cluster represents one protein. Blue lines correspond to proteins with a strong fit to the cluster centroid, while green and yellow lines represent proteins with progressively weaker associations peripheral to the cluster centroid. The age groups of men are young adult (21–30 years), late adult (31–40 years), and advanced age (41–51 years).

Fuzzy c-mean clustering of 588 total proteins identified nine clusters based on expression changes across groups (Figure 6A). Proteins with significant changes are shown as blue lines, totaling 207 (Figure 6B). These dynamic variations may reveal age-related biomarkers, particularly evident in distinct expression patterns within clusters 2 and 3 for the young adult group, clusters 4 and 9 for the late adult group, and clusters 6 and 8 for the advanced age group. Table 2 offers a summary of the functional analysis of the DEPs, highlighting significant enrichments for “acetylation” and “phosphoprotein” in the young adult group (Table 2A), “extracellular exosome” and “acetylation” in the late adult group (Table 2B), and “extracellular exosome,” “acetylation,” and “mitochondrion” in the advanced age group (Table 2C). Additionally, Table 2 lists highly significant DEPs compared to those in the advanced age group. Further details of the distribution of proteins in various clusters are provided in Supplementary Data File S1.

## 3. Discussion

Sperm quality in men declines with age, impacting their fertility potential. While many studies have examined changes in sperm over time, there is limited research on the functional genomics of sperms, particularly proteomics. This is a novel age-associated proteomic study on the spermatozoa of normal, fertile men. This study uses high-throughput LC-MS/MS techniques to profile sperm proteomes in three stratified age groups of fertile men: young adult (21 to 30 years), late adult (31 to 40 years), and advanced age (41 to 51 years old). Notably, significant changes in protein abundance were found in the advanced age group, suggesting potential effects on fertility outcomes.

### 3.1. Semen Characteristics

The three age groups of men in this study differed from each other in their mean age. The mean sperm concentrations were lower in the young adult group compared to the late adult group but were comparable to those in the advanced age group. Other semen parameters showed no significant differences across the groups. Previous research indicates that men over 40 generally have lower sperm concentrations and less favorable semen analysis results [1,7,32,37,53,54,55]. A recent study found that men aged 21–30 had lower sperm concentrations than those aged 31–50; however, concentrations in men over 50 were similar to those in the younger age group [6]. Our findings support these mixed reports, showing no significant differences in sperm concentrations or parameters between the young and advanced age groups.

### 3.2. Spermatozoal Proteome

Global proteome analyses of spermatozoa in this study identified 588 unique proteins, showing, on preliminary observation, a notable age-dependent decline across different groups: young adults had 581 proteins, late adults had 570, and the advanced age group had 562. This decline in sperm proteins with age has not been documented before. Possible reasons include impaired spermatogenesis in aged testes, reduced binding of testicular proteins, or decreased secretion from the epididymis and accessory glands associated with the aging process [34,56]. Research on age-related sperm proteomes is limited. To our knowledge, only two proteomic studies have investigated this important research area, both of which exhibit notable limitations in their study designs [52,53]. The first study compared the sperm proteomes of younger fathers (aged 28–32 years) with those of older fathers (aged 68–72 years) [52]. A total of 22 protein spots with significantly different densities were extracted and analyzed using 2-D gel electrophoresis, in conjunction with matrix-assisted laser desorption/ionization time-of-flight mass spectrometry (MALDI-TOF MS). This study’s limitations include its inability to capture the full spectrum of proteins present in sperm and its exclusive focus on a single protein, prostate and testis expressed 1 (PATE1). Additionally, the older participant group comprised elderly men who were beyond the typical reproductive age, which limits the potential for meaningful comparisons with the older group in our current study. In the second study [53], 12 sperm samples were analyzed for age-related proteomic changes across two age groups (26–30 years and 45–54 years), identifying a total of 2369 proteins, with 80 showing significant alterations (64 upregulated and 16 downregulated) in the older group; however, the participants were men of unknown fertility status who may have been seeking fertility consultation, which introduces the possibility that compromised fertility could have confounded the proteomic results due to factors beyond aging, thus complicating comparisons.

Recent studies have extensively explored the proteomic profile of human sperm, revealing protein counts ranging from 75 to 6238 [40,43,50,57]. Variations in these counts arise from different technical platforms, clinical conditions, and infertility phenotypes. For example, 75 proteins were identified in normozoospermic men [57], whereas counts reached 1139 and 1095 in normozoospermic fertile men and infertile men, respectively [48]. In addition, 667 proteins were identified in asthenozoospermic men [58], 933 proteins were found in men with unilateral varicocele and 1230 in those with bilateral varicocele [59], and 5778 proteins were identified in a diverse cohort of fertile and infertile men [50]. Variations in protein detection across studies can result from differences in sample preparation, protein extraction and digestion protocols, purification techniques, and the mass spectrometry instruments used. Databases, algorithms, and data filtering criteria also influence outcomes. In this study, we required at least three unique peptides per protein and set a false detection rate (FDR) of less than 1% to ensure high-confidence protein identification.

### 3.3. Proteins Specific to Age Groups

Of the 588 proteins identified across the three age groups, about 93% were present in all the three groups. These represent the core proteins essential for the structure and function of human spermatozoa, including morphology, motility, and sperm–egg interaction [40,43]. Interestingly, more proteins were uniquely expressed (5 proteins) or missing (26 proteins) in the advanced age group compared to the other two groups. Seven proteins were specific to individual age groups, potentially serving as age-related biomarkers. MYLK4, a protein kinase present only in the young adult group, is involved in the phosphorylation and signaling pathways in skeletal muscle [60,61]. A recent study reported downregulation of the MYLK4 gene in the muscles of aged rats [62], but its role in sperm remains unstudied. Similarly, PRSS57 was detected only in the spermatozoa of men in the late adult group. PRSS57 is a trypsin-fold protease that cleaves after arginine residues and is involved in peptidyl-serine phosphorylation [63]. It regulates immune responses [64] and defends against pathogens [65]. The detection of PRSS57 in the spermatozoa of late adult men suggests a potential, yet unexplored, role in sperm function related to age. One possible speculation could be its role in the acrosome reaction through interaction with SPINK2, a serine protease inhibitor [66]. PRSS57 is a target protease for SPINK2 in hematopoietic stem and progenitor cells. SPINK2 inhibits acrosin, which is involved in the acrosome reaction in spermatozoa. SPINK2 was consistently expressed in sperms across age groups in the present study; its abundance increased with age, though not significantly. Notably, mutations in SPINK2 are associated with oligospermia and male infertility [66]. Given its expression pattern and potential link to SPINK2, further research is needed to elucidate the role of PRSS57 in sperm and its relevance to age-related fertility changes.

The advanced age group revealed five specific proteins—KRT4, MGRN1, LPGAT1, HMGB4, and OXCT2—whose roles in older male spermatozoa merit further investigation. Age-associated increased KRT4 mRNA has been linked to prostate cancer [67]; however, its role in sperm function is unknown. MGRN1, a RING-type E3 ligase, is selectively expressed in the epididymal spermatozoa of older dogs [68], while in mice, its expression increases in the testes with age [69]. A deficiency in MGRN1 in mice leads to decreased hormone levels, reduced sperm concentration, and sterility, negatively impacting mitochondrial health [70]. LPGAT1 plays a crucial role in glycerophospholipid remodeling [71]. In LPGAT1-overexpressing mice, a shift in Ca^2+^ handling from the fast-twitch to slow-twitch muscle phenotype occurs [72], but the importance of this function in the sperm remains unexplored. Downregulation of LPGAT1 in an in vitro model improved mitochondrial function [73]. Conversely, deletion of LPGAT1 in zebrafish results in impaired sperm motility and developmental issues [74]. Additionally, the age-related downregulation of LPGAT1 in avian sperm storage may diminish reproductive efficiency [75]. HMGB4, a transcriptional repressor in the adult mouse testis, is present in round and elongating spermatids, but not in later spermatozoa stages [76]. It is abundant in bull and stallion spermatozoa, and its upregulation has been associated with poor-quality sperm [77]. OXCT2 is essential for ketone body metabolism, exhibiting testis-specific mRNA and localized presence in sperm midpieces [78]. Its expression increases under oxidative stress in human sperm [79] and is observed in bulls with lower fertility [80]. However, OXCT2 deletion in mice does not impact fertility [81], warranting further exploration.

Among the 24 proteins present in both young adult and late adult groups, but absent in the advanced age group, are MRTO4, CPNE2, and ANXA13, which are involved in ribosome assembly, cellular growth, and signal transduction [82,83,84]. Additionally, proteins such as CISD1 and SLC25A19 play essential roles in energy metabolism, particularly electron transport and oxidative phosphorylation [85]. Other important proteins for sperm motility and microtubule organization include PGK2, CFAP161, and CETN1 [86,87,88]. Proteins such as RPS16 and SQLE are involved in catalytic activity [89,90]. Notably, SQLE is an essential enzyme in cholesterol synthesis. Cholesterol is a critical substrate for steroid hormones and a constituent of cell membrane architecture [90]. Non-detection of SQLE in advanced-age men may be associated with defective testosterone synthesis, which is critical for spermatogenesis [90]. Overall, the low expression or absence of the above proteins in the spermatozoa of men in the advanced age group suggests potential functional deficits that may affect critical sperm functions.

Of the top 20 abundant proteins in each of the three age groups, 14 of the 33 proteins were associated with individual age groups. Semenogelin-2 (SEMG2) and putative tripartite motif-containing protein 61 (TRIM61) were highly abundant proteins in the young adult group and the late adult group, respectively. SEMG2 is believed to be involved in semen coagulum formation; however, its specific role in sperms remains unknown [47]. Downregulation of SEMG2 occurs under high oxidative stress [91], and interestingly, SEMG2 was downregulated in the advanced age group in the present study. Conversely, upregulation of SEMG2 in older men has also been reported [53]. TRIM61 has been reported to be associated with pre-implantation development in mouse embryos and in other cellular processes [92]. For the advanced age group, the top abundant proteins included cell signaling glycoprotein Wnt-2 (WNT2), which is associated with testicular morphology and sperm motility [93] and neurodegenerative diseases such as Alzheimer’s disease [17]. Semenogelin-1 (SEMG1) is involved in coagulum formation, sperm survival and protection, and the inhibition of capacitation [94,95]. Proteasome subunit beta type-3 (PSMB3) is involved in intracellular proteolysis activity [96] and sperm morphogenesis and function [48]. The 26S proteasome regulatory subunit 8 (PSMC5) is involved in maintaining cellular protein homeostasis and DNA damage repair activities [97]. Among the highly abundant proteins common to the three groups, A-kinase anchor protein 4 (AKAP4) and glutathione peroxidase 4 (GPX4) were significantly downregulated in the advanced-age men compared to the other two groups. AKAP4 and AKAP3 are the two main abundant structural components of the fibrous sheath around the axoneme, providing mechanical support to the sperm flagellum [98,99]. AKAP4 contains anchoring sites for cAMP-dependent protein kinases, which facilitate the localization and enhancement of tyrosine phosphorylation of sperm proteins, initiate signal-transduction processes, and regulate flagellar activity. AKAP4-null mice are infertile and exhibit reduced sperm numbers, dysplasia of the fibrous sheath, and shortened flagellum, all of which contribute to a lack of progressive motility. This deficiency arises due to the failure of signal transduction and glycolytic enzymes to interact appropriately with the fibrous sheath [98]. GPX4 is an antioxidant protein that protects sperm cells from oxidative damage, ensuring the structural integrity of sperm chromatin [100]. Taken together, compared to young adult and late adult men, the absence of SEMG2 and TRIM61 within the abundant proteins in the advanced age group may compromise protein homeostasis, gene regulation in sperm, and early embryo recognition and development. A high abundance of WNT, SEMG1, PSMB3, PSMC5, and other proteins in the advanced age group may be associated with risk for neurodegenerative diseases, malignancies, and impact sperm DNA integrity, spermatozoa survival and function, and fertilization. The downregulation of AKAP4 and GPX4 in the advanced age group may be associated with declining sperm motility and reduced antioxidant activity with aging. In summary, the differential presence, absence, or abundance of specific proteins in the spermatozoa of the advanced age group is potentially associated with their altered functional landscape, lending support to the role of sperm proteome in age-associated impairments in male fertility. Additional investigations using larger sample sizes, quantitative biochemical analyses, targeted proteomic approaches, and functional validation assays are crucial for confirming the observed associations and clarifying their biological and clinical significance.

In the reported aging-associated sperm proteome study on men with unknown fertility status [53], although 80 proteins were differentially expressed, only four proteins were highlighted in the report. Of the four proteins, SEMG2 and clusterin (CLU) were upregulated, and testis-specific H1 histone (H1-7) and cilia- and flagella-associated protein 44 (CFAP44) were downregulated in the aged men. In contrast, in the current study, SEMG2 was downregulated in the aged men, CLU and H1-7 were not differentially expressed, and CFAP44 was not detected.

### 3.4. Functional Enrichment and Key Aging-Related Proteins

Gene ontology analyses indicate notable enrichment in extracellular proteins related to enhanced secretion by the epididymis and accessory sex glands or increased sperm binding during and post-ejaculation [101]. These proteins are crucial for sperm maturation, capacitation, acrosome reaction, and interactions with the oocyte [102,103]. The decline in protein enrichment in older men in the advanced age group is likely due to age-related reductions in contributions from the epididymis, seminal vesicle, and prostate [34]. Aging also affects biological processes, increasing protein folding and proteasomal protein catabolism while decreasing flagellated sperm motility and glycolysis. Proper protein folding is crucial for normal function, and disruptions during spermatogenesis can result in defective proteins and impaired sperm functionality [104]. Additionally, proteasomal proteins are essential for degrading proteins involved in spermatogenesis and chromatin remodeling [96].

Functional analysis revealed reduced levels of PTMs in the advanced age group, including acetylation, phosphorylation, and methylation. PTMs are essential for regulating protein stability and function, particularly in spermatozoa, which have limited translational and transcriptional activities [105,106]. Notably, tyrosine phosphorylation is critical for spermatogenesis, motility, and the acrosome reaction [106,107]. Deficiencies in phosphoproteins linked to sperm motility and fertilization have been identified in low-motility sperm [108]. Additionally, oxidative PTMs, such as thiol oxidation and tyrosine nitration, are vital for sperm fertilization ability [109]. Age-related oxidative stress may negatively impact sperm motility, capacitation, and DNA integrity [1,5]. Acetylation plays a crucial role in various metabolic processes during spermatogenesis [110,111]. Overall, the deregulation of PTMs in the older age group may be associated with the disruption of sperm functions and impaired fertility.

KEGG pathway analyses indicated significant enrichment in key pathways linked to neurodegenerative diseases, such as Parkinson’s, prion disease, Huntington’s, and Alzheimer’s, in men in the advanced age group. Notably, 60% of the top enriched pathways were associated with these diseases, suggesting a connection between neurodegenerative pathways and sperm function with advancing age. Conversely, pathways related to glycolysis/gluconeogenesis and carbon metabolism showed decreased enrichment in the advanced age group. These pathways are crucial for cellular functions, including nucleotide synthesis and DNA protection [112]. Their decreased levels have been linked to male infertility, given their importance for sperm energy metabolism, which supports motility and fertilization [113,114]. These preliminary insights into age-associated proteomic alterations highlight potential overlaps in molecular and biological pathways, as identified by functional enrichment analysis. Furthermore, we emphasize the critical need for future studies involving larger, clinically stratified cohorts, coupled with rigorous quantitative biochemical assays, targeted proteomic strategies, and functional validation experiments.

The age-group cluster analysis identified proteins potentially characteristic of the young adult group within Clusters 2 and 3. Notable proteins include histone H2B type 1 (H2BC1), which is crucial for spermiogenesis [115]; cytosol aminopeptidase (LAP3), which aids in cell proliferation [116]; and protein disulfide isomerase A4 (PDIA4), essential for spermatogenesis [117]. Other important proteins are lactotransferrin (LTF), known for its antioxidant properties [45], and dynein light chain Tctex-type protein 2 (DYNLT2), which contributes to sperm motility [118]. Among the proteins characteristic of the late adult group within Clusters 4 and 9 are meiotic recombination protein (SPO11), which is required for initiation of meiotic recombination and chromosomal synapsis [119]; ATP synthase subunit beta (ATP5F1B), which is a critical subunit of ATP synthase essential for increasing ATP levels [120]; and testis-expressed protein 45 (TEX45), which stabilizes sperm axonemal microtubules [121]. Other important proteins include calcium-binding protein 5 (CABP5), a crucial component of calcium-mediated cellular signal transduction; MGRN1; and serine/threonine-protein kinase B-Raf (BRAF), which is involved in the mitogen-activated protein kinase pathway [122].

Several proteins in Clusters 6 and 8 are identified as potential markers for the advanced age group. Six proteins—Ras-related protein Rab-15 (RAB15), septin-4 (SEPTIN4), sperm equatorial segment protein 1 (SPESP1), ubiquitin carboxyl-terminal hydrolase 5 (USP5), annexin A1 (ANXA1), and peroxiredoxin-1 (PRDX1)—showed decreased expression in this group compared to the young age group. RAB15 is involved in membrane trafficking; however, its role in sperm function remains unclear [123]. Overexpression of RAB15 was reported in subfertile bull spermatozoa [124], warranting further sperm-related investigations. SEPTIN4, a critical component of the sperm annulus, is essential for motility [125]; male mice lacking it are sterile and exhibit abnormal sperm morphology [125,126]. SEPTIN4 is vital for maintaining mitochondrial architecture during spermiogenesis, and its deregulated expression in human sperm has been linked to infertility [123]. Additionally, SEPTIN4 is crucial for sperm capacitation [125]. The absence of SEPTINA4 in the sperm of men in the advanced age group, as observed in this study, may on a preliminary basis reflect age-related probable deficits in morphology, motility, and capacitation.

SPESP1 is essential for sperm fertilization and equatorial segment architecture [127]. It is detected in the testis during various stages of sperm development [127,128,129]. In mice, SPESP1 is crucial for sperm fusion; its absence leads to fewer pups and delayed fertilization [48,128,130] and results in the loss of the equatorial segment membrane [128]. A review highlighted that SPESP1 levels were downregulated in poor-quality semen [131]. Additionally, a study found reduced SPESP1 protein and mRNA levels in the skin tissue of aged mice and humans [132]. The interesting age association of SPESP1 was demonstrated by its deletion, which delayed wound healing in young mice, whereas its overexpression improved healing in older mice. It is suggested that downregulation of SPESP1 in older men may be associated with impaired sperm–oocyte fusion and compromised fertility.

USP5 is a ubiquitin-specific protease involved in various cellular functions, including sperm development [133,134]. It regulates the protein phosphatase 2 scaffold subunit Abeta (PPP2R1B), whose deficiency impairs meiotic recombination in mice and humans. ANXA1 is a Ca^2+^-dependent membrane-binding protein with immunomodulatory functions, found in spermatozoa, particularly in the sperm head, mid-piece, and flagellum [135]. Lower ANXA1 levels correlate with poor semen quality and high DNA fragmentation. Additionally, ANXA1 facilitates sperm binding to the cow oviduct [136]. The current study suggested that the downregulation of ANXA1 in the spermatozoa of advanced-age men may be associated with diminished sperm binding and an altered spermatogenic environment impacting fertility. PRDX1 is a thiol-specific antioxidant enzyme that is essential for protecting sperm from oxidative stress, with reduced levels being linked to infertility [137,138,139]. It is predominantly expressed in various spermatogenic cells and spermatozoa [140]. Date palm pollen has been shown to improve semen quality and enhance PRDX1 expression in infertile men [141]. In the current study, PRDX1 levels were lower in the spermatozoa of men in the advanced age group, contrasting with findings showing increased levels in germ cells of older mice, which were attributed to compensatory protective mechanisms [142].

The current study does not present serum endocrine data (e.g., FSH, LH, and testosterone), which previous research has indicated to have unfavorable correlations with the aging of men [1,10,143]. Additionally, the pooling strategy employed in this study is a cost-effective method designed to minimize individual variability within age groups while preserving significant variability between groups. This strategy enhances the sensitivity of protein detection, thus improving the accuracy of fold change estimation. It is essential to note that this approach considers the potential trade-offs associated with the loss of individual information, which can impact statistical inference. Nevertheless, this exploratory study, which utilizes independently pooled samples and implements appropriate statistical analyses, includes adjusted *p*-values, the Hochberg–Benjamini procedure, and the false discovery rate to ensure robust conclusions. Collectively, these limitations highlight the need for large-scale validation of the differentially expressed proteins identified as potential markers for the effects of male aging on the sperm proteome, which could facilitate future therapeutic interventions.

Overall, the study explored a range of differentially expressed proteins that may potentially differentiate aging spermatozoa from those of young adult men. Specifically, the study observed age-associated alteration across multiple domains crucial to sperm physiology and fertility: (1) spermatogenesis, reflected by alterations in proteins such as MGRN1, LPGAT1, PSMB3, H2BC1, LAP3, PDIA4, SPO11, USP5, CPNE2, MRTO4, and HMGB4, suggesting impaired sperm development and maturation; (2) sperm motility, suggested by changes in proteins such as LPGAT1, PGK2, CFAP161, CETN1, WNT2, AKAP4, DYNLT2, TEX45, and SEPTIN4 that are critical for flagellar movement; (3) energy metabolism, represented by alterations in OXCT2, CISD1, SLC25A19, and ATP5F1B associated with ATP production and mitochondrial function, compromising sperm viability and performance; (4) cellular homeostasis and DNA damage repair, as reflected by alterations in PSMC5 that may compromise the ability of sperm to maintain proteostasis and genomic integrity; (5) antioxidation, suggested by changes involving GPX4, LTF, and PRDX1 pointing to a diminished defense against oxidative stress; (6) immune response, reflected by changes in ANXA1 and ANXA13, suggesting age-related modulation of sperm immunological signaling; (7) catabolism, reflected by alterations in RPS16 and SQLE, highlighting biosynthetic and degradation pathways that could compromise sperm structural integrity; and (8) capacitation, acrosome reaction, and fertilization, illustrated by alterations in SEMG1, SEMG2, CABP5, and SPESP1, which may potentially impair the fertilization efficiency of sperms. In addition, several proteins with currently unknown sperm-specific functions such as KRT4, PRSS57, RAB15, and MYLK4 were also found to be dynamically regulated with age, suggesting their possible role in age-related reproductive decline in men. Collectively, these proteomic alterations in spermatozoa from aging men, on a preliminary basis, reflect a multifactorial disruption of the key processes essential for fertility.

## 4. Materials and Methods

### 4.1. Study Design and Sample Collection

The research protocol for this study was approved by the Center for Excellence in Genomic Medicine and Research (CEGMR) Bioethics Committee of King Abdulaziz University, Jeddah, Saudi Arabia (approval code 11-CEGMR-Bioeth-2020, dated 18 December 2020). The study included 24 healthy men of Arab/Saudi ethnicity. Each participant signed an informed consent form before being included in the study. The men were between 21 and 51 years old, married, and fertile, each having naturally fathered at least one child (irrespective of the criterion of birth within the preceding 12 months) before enrolling in the study. All the recruited men underwent a clinical assessment, which included a detailed personal and family history, as well as a physical examination for reproductive tract abnormalities at the Department of Urology, King Abdulaziz University Hospital. Each recruited man donated an entire semen sample in a sterile container by masturbation after 3–5 days of sexual abstention at the King Fahd Medical Research Center or Center for Innovation in Personalized Medicine, King Abdulaziz University. The semen sample was immediately transferred to the laboratory. Exclusion criteria for men included a history of genetic or congenital disorders, abnormalities of the reproductive tract, recent genitourinary infection or inflammation, metabolic syndrome, chemotherapy (or radiotherapy), acute or chronic diseases, or abnormal semen analysis. Six of the twenty-four men were excluded based on low spermatozoa progression (two samples), low normal spermatozoa forms and progression (one sample), technical error (one sample), low normal spermatozoa forms, and unproven fertility (no kids; one sample) and previous unilateral orchiectomy (one sample), resulting in the inclusion of 18 semen samples in the current study. Finally, semen samples from 18 men in three groups according to the age of men were as follows: young adult group (21–30 years; *n* = 6), late adult group (31–40 years; *n* = 7), and advanced age group (41–51 years; *n* = 5). The age group classification was based on the available literature and our focus on fertility decline with age. The three age groups represent the most reproductively active age decades in men. In this regard, the percentages of infants born to fathers aged >40, >50, >60, and >70 years were approximately 9%, 0.9%, 0.1%, and 0.008%, respectively [19,144]. The young adult group (21–30 years) and the late adult group (31–40 years) account for the majority of contributions to fatherhood percentages. The age of 41–51 years in men is considered advanced parental age. Several studies have reported the age of around 40 years as the onset of changes in semen quantity and quality, as well as fertility decline in men [1,2,7,32,37].

### 4.2. Semen Analysis, Processing, and Sperm Purification

Immediately upon arrival in the laboratory, the semen samples were incubated for 30 min at 37 °C to facilitate liquefaction. The semen samples were analyzed according to the guidelines of the World Health Organization [145]. A gross examination of the semen samples was done for volume, pH, consistency, and color. Micro-examination of the semen samples included sperm concentration, motility, progression, morphology, total sperm count per ejaculate, total live sperm per ejaculate, and morphology using a Makler counting chamber (Origio-Cooper Surgical, Ballerup, Denmark) or a glass slide. Other somatic/round cells and agglutination were also assessed.

The semen samples were centrifuged at 1000× *g* for 10 min to separate spermatozoa from seminal plasma. Sperm pellets were re-suspended in 2 mL of PBS. The density gradient centrifugation method was used to separate the active spermatozoa. This included two layers of gradient solution with a lower layer of 1 mL of 80% and an upper layer of 1 mL of 40% PureCeption (Cooper Surgical, Ballerup, Denmark) gently overlaid with 1 mL of sperm suspension in a 15 mL conical tube and centrifugation at 500× *g* for 10 min. After carefully aspirating the gradient and dead cell layers, the resulting pellet (approximately 100 µL) was resuspended in 6 mL of PBS. The purified sperm cells were counted using a Makler counting chamber and then centrifuged at 1000× *g* for 10 min. The supernatant PBS was removed, and the final sperm pellet was supplemented with the complete™ EDTA-free Protease Inhibitor (catalog# 11873580001, Sigma Aldrich, Darmstadt, Germany) per the manufacturer’s recommendation. Each sperm pellet was aliquoted, labeled, and stored at −80 °C until proteomic analysis.

### 4.3. Proteome Analysis

#### 4.3.1. Sperm Cell Preparation and Protein Extraction

The sperm samples were thawed on ice (4 °C). Each sperm sample was lysed using 0.1% RapiGest SF lysis buffer (Waters, Manchester, UK), which was prepared in 50 mM ammonium bicarbonate and supplemented with protease inhibitors phenylmethylsulphonyl fluoride (20 µM) and benzamidine (830 µM). After cell lysis, the samples were centrifuged at 13,000 rpm for 30 min at 4 °C to collect the total proteins in the supernatants. Protein samples were quantified using the Bradford assay method. Due to the limitation of the analytical multiplexing ability of LC-MS/MS analysis, the protein samples were pooled based on age, generating three age groups each containing an equal amount of protein (50 µg) for in-solution digestion.

#### 4.3.2. In-Solution Protein Digestion

This procedure was executed as previously reported [146,147]. Briefly, each protein sample was exchanged twice with 500 μL of 0.1% RapiGest (Waters, Manchester, UK) using a 3-kDa ultrafiltration device (Millipore, Bedford, MA, USA), leading to a final protein concentration ranging from 0.1 to 1 μg/μL. Total proteins in all sample groups were denatured at 80 °C for 15 min, then reduced in 10 mM dithiothreitol (DTT) at 60 °C for 30 min. The samples were cooled down to room temperature and then alkylated in 10 mM iodoacetamide (IAA; 1.0 µL/10 µL) in the dark for 40 min with gentle agitation. The temperature difference between the reduction and the addition of IAA minimizes the quenching effect of DTT on the added IAA. The resulting protein mixtures were trypsin-digested (enzyme–protein ratio, 1:50 *w*/*w*; 1 μg/μL trypsin; Promega, Madison, WI, USA) overnight at 37 °C with gentle shaking. The reaction was stopped with 12 M HCl, and the extracted samples were concentration adjusted with aqueous 0.1% formic acid to achieve concentrations of 1 μg/μL of digested peptides before to LC-MS/MS analysis. All sample groups were processed simultaneously in a single batch.

#### 4.3.3. Protein Identification Using Mass Spectrometry-LC-MS/MS

Quantitative profiling of the in-solution-digested proteins in sperm cell lysate pools was performed using label-free one-dimensional nano acquity liquid chromatography coupled with tandem mass spectrometry on a Synapt G2 HDMS on a Trizaic Nano-Flow source (Waters, Manchester, UK), as previously described [146,147]. The settings for the instrument for electrospray ionization mass spectrometry analyses were optimized on the tune page by setting up detectors using 2 ng/μL leucine enkephalin (556.277 Da), and mass (*m*/*z*) calibration was achieved on a separate infusion of 500 fmol [Glu] 1-fibrinopeptide B (GluFib, 785.843 Da; Waters, Manchester, UK) using the Mass Lynx IntelliStart (Waters, Manchester, UK). Other instrument conditions included 3 kV capillary voltage, 50 V sample cone, 5 V extraction cone, 80 °C source temperature, 10 L/h cone gas, 0.5 bar nano flow gas, and 800 L/h purge gas. All analyses were carried out on Trizaic Nano source (Waters, Manchester, UK) ionization in the positive ion mode nanoESI. A total of 3 μg of each peptide digest of pools of sperm cell lysates prepared above was loaded on a C18 trap column (180 μm × 20 mm, 5 μm) and an HSS T3 analytical column (85 μm × 100 mm, 1.8 μm). All samples were processed using the equity sample manager with a mobile phase consisting of A1 (99% water:1% acetonitrile:0.1% formic acid) and B1 (100% acetonitrile:0.1% formic acid) with a sample flow rate of 0.5 μL/min. Data independent acquisition (MSE) experiments were performed, and data was acquired over a range of *m*/*z* 50–2000 with 0.9 s scan time, 20–50 V ramped transfer collision energy, and 120 min total acquisition time using ion-mobility separation experiments (HDMS). Complex protein profiles of the in-solution digested pool samples were generated. All peptide digest pool samples were analyzed in triplicate runs to ensure technical reproducibility and to reduce instances of missing values in the generated datasets. The data were acquired using the Mass Lynx program (version. 4.1, SCN833; Waters) operated in the resolution and positive polarity modes. The acquired mass spectrometry data were background-subtracted, smoothed, and de-isotoped at a medium threshold. The data processing and database searching was done using Progenesis QI for proteomics (QIfp) v3.0 (Waters/Nonlinear Dynamics, Newcastle, UK). The generated peptide masses were searched against the protein sequence database UniProt (https://www.uniprot.org/), accessed on 22 December 2023, using Progenesis LC/MS (QIfp, v3.0) (Nonlinear, Newcastle, UK).

### 4.4. Statistical Analysis, LC-MS/MS Data Analysis, and Bioinformatics

The group effect on semen parameters was evaluated using one-way ANOVA, followed by Tukey’s test for group comparisons when applicable, with *p* < 0.05 considered as significant. LC-MS/MS data processing was performed using an in-built algorithm in Progenesis QI for proteomics (QIfp v3.0; Waters/Nonlinear Dynamics, Newcastle, UK). The peptide ion list was queried in the UniProt/SwissProt (https://www.uniprot.org/) using criteria such as one missed cleavage, a minimum of 3 unique peptides per protein, a maximum protein mass of 1000 kDa, and a false discovery rate (FDR) of ≤1%. Further analysis included a normalized label-free Hi3 algorithm in Progenesis QI software (QIfp) v3.0. In Progenesis QI for proteomics, the Hi-N (Hi3) normalization method is implemented following the peptide and protein identification steps in Progenesis. Each peptide’s abundance is calculated from all its constituent peptide ions. For each protein, the N most abundant peptides have their abundances averaged to provide a measure of the protein’s signal. This approach allows for the relative quantitation of the same protein across runs of the internal spiked protein standard, alcohol dehydrogenase (ADH, P003300). The relative quantitation was then converted to an absolute measurement of the protein amount in Progenesis QI for proteomics. Proteins with marked differences in their abundance were considered as DEPs between age groups and were identified by filtering the proteome for significant difference (*p*-value < 0.05) and at least >1.5-fold in altered abundance. Trans Omics software (Nonlinear Dynamics, Newcastle, UK) was used for unsupervised principal component analysis (PCA) and hierarchical clustering for the three age groups. Venn diagrams were used to analyze the distribution of proteins among the groups (https://bioinformatics.psb.ugent.be/webtools/Venn/ [accessed on 22 June 2025]). Ontological classification and functional analyses were conducted using DAVID bioinformatic tools (https://davidbioinformatics.nih.gov/summary.jsp [accessed on 22 June 2025]), and significance was determined using the Benjamin–Hochberg method and FDR-adjusted *p*-values of less than 5%. To examine the time-series evolution of protein expression patterns, fuzzy C-means clustering was applied using Cytoscape (https://cytoscape.org/ [accessed 22 June 2025]) [148]. This unsupervised method groups similar data points and assigns each data point a likelihood or probability score for being in that cluster, such that the data points in the same cluster are identical to each other, and data points in different clusters are dissimilar. Mean sperm protein expression values by age group were input into the algorithm, generating nine soft clusters. The expression patterns were represented by colored trend lines and characterized by the fluctuation of proteins across the young adult group, the late adult group, and the advanced age group. Each line corresponds to a single protein. Blue lines indicate proteins with a strong fit to the cluster centroid, while green and yellow lines represent proteins with progressively weaker associations peripheral to the cluster centroid.

## 5. Conclusions

This study highlights the dynamic proteomic changes in spermatozoa associated with aging in men. The understanding of how aging impacts sperm fertility remains limited, but it is vital for addressing fertility challenges in aging men. In this study, we conducted a proteomic analysis using LC-MS/MS on spermatozoa from healthy, fertile men categorized into three distinct age groups: young adult, late adult, and advanced age. A total of 588 proteins were identified, with the number decreasing with age. Notably, the advanced age group exhibited a reduced number of proteins compared to the other two groups. Functional analyses revealed significant shifts in enriched gene ontology (GO) terms, indicating increased associations with neurodegenerative diseases and protein folding, as well as reductions in glycolysis, flagellated sperm motility, and antioxidation processes. Cluster analysis corroborated these dynamic proteomic changes, emphasizing key proteins that may be essential for understanding how aging affects sperm function and fertility. Specifically, significant alterations were observed in proteins related to spermatogenesis, motility, energy metabolism, cellular homeostasis and DNA damage repair, antioxidation, capacitation, acrosome reaction, fertilization, and embryo development. Collectively, these findings demonstrate that advancing paternal age is associated with extensive proteomic remodeling in spermatozoa, reflecting multifaceted impairments in biological processes critical for sperm function and reproductive competence. Proposed combinations of specific proteins could serve as potential indicators of spermatozoa from young adults (LTF/DYNLT2 versus H2BC1/SQLE), late adults (SPO11/TEX45 versus BRAF), and men of advanced age (ANXA1/SEPTIN4/RAB15 versus SPESP1). Furthermore, this study emphasizes the necessity for additional research to validate these proteins as biomarkers of male reproductive aging, investigate their mechanistic roles in sperm function and fertility, and develop therapeutic strategies to mitigate age-related reproductive decline in men.

## Figures and Tables

**Table 1:** Semen analysis endpoints in the three age groups of men. The age groups of men are young adult (21–30 years), late adult (31–40 years), and advanced age (41–51 years). Data are expressed as means (±SD); overall *p*-value is from one-way ANOVA; different superscripts (^a–c^) indicate significant differences (*p* < 0.05) by Tukey’s test; Sperm conc. = Sperm concentration; Total sperm/Ejac. = Total sperm/Ejaculate.

Endpoints	Young Adult Group (*n* = 6)	Late Adult Group (*n* = 7)	Advanced Age Group (*n* = 5)	*p*-Value
Age (years)	27.8 ± 2.6 ^a^	34.3 ± 2.9 ^b^	47.2 ± 4.2 ^c^	0.001
BMI (kg/m^2^)	28.68 ± 3.75	26.33 ± 2.88	25.90 ± 2.05	0.114
Abstinence (days)	4.2 ± 0.48	4.1 ± 2.0	3.6 ± 0.9	0.489
Total Volume (mL)	3.33 ± 1.84	2.47 ± 0.80	5.10 ± 2.79	0.141
Sperm conc. (×10^6^/mL)	55.3 ± 25.54 ^a^	85.4 ± 22.06 ^b^	78.0 ± 38.24 ^ab^	0.022
Total motility (%)	63.17 ± 17.91	69.86 ± 9.06	67.40 ± 12.88	0.201
Progression (%)	39.83 ± 10.44	45.43 ± 13.79	48.40 ± 12.26	0.217
Total sperm/Ejac. (×10^6^)	194.33 ±18.21	217 ± 11.36	337.90 ± 18.71	0.396
Total motile sperm (×10^6^)	142.05 ± 19.21	153.44 ± 84.93	220.65 ± 115.68	0.443
Normal morphology (%)	4.5 ± 0.55	6.0 ± 3.61	5.4 ± 1.14	0.169

**Table 2:** Functional analysis and selected differentially expressed proteins in age-related clusters.

**(A) Key Enriched Biological Functions**		**Selected DEPs in Young Adult Group (YA)**		
**Categories**	**FDR**	**Proteins**	***p*-Values**	**YA/AA**
kw-0007~Acetylation	4.2 × 10^−3^	Squalene monooxygenase (SQLE)	9.5 × 10^−10^	ND in AA
Kw-0597~Phosphoprotein	1.3 × 10^−2^	Histone H2B type 1 (H2BC1)	8.4 × 10^−6^	982.5
GO:0005739~mitochondrion	2.1 × 10^−3^	Cytosol aminopeptidase (LAP3)	4.2 × 10^−4^	10397
		Protein d. isylfide-isomeraseA4 (PDIA4)	1.4 × 10^−4^	12.6
		Lactotransferrin (LTF)	3.5 × 10^−4^	75.9
		Dynein light chain Tctex-type protein 2 (DYNLT2)	5.4 × 10^−3^	272.6
**(B) Key Enriched Biological Functions**		**Selected DEPs in Late Adult Group (LA)**		
**Categories**	**FDR**	**Proteins**	***p*-Values**	**LA/AA**
GO:0070062~extracellular exosome	9.2 × 10^−6^	Meiotic recombination protein (SPO11)	2.4 × 10^−11^	ND in AA
KW-0007~Acetylation	2.3 × 10^−4^	ATP synthase subunit beta (ATP5F1B)	1.5 × 10^−6^	12674.5
GO:0005634~nucleus	2.8 × 10^−2^	Testis-expressed protein 45 (TEX45)	4.1 × 10^−4^	5250.2
hsa01200:Carbon metabolism	2.8 × 10^−2^	Calcium-binding protein 5 (CABP5)	2.3 × 10^−7^	0.5
KW-1017~Isopeptide bond	3.3 × 10^−2^	E3 ubiquitin-protein ligase MGRN1 (MGRN1)	1.0 × 10^−6^	ND in LA
		Serine/threonine-protein kinase B-raf (BRAF)	3.42 × 10^−3^	0.3
**(C) Key Enriched Biological Functions**		**Selected DEPs in Advanced Age Group (AA)**		
**Categories**	**FDR**	**Proteins**	***p*-Values**	**AA/LA**
GO:0070062~extracellular exosome	4.6 × 10^−7^	Ras-related protein Rab-15 (RAB15)	1.1 × 10^−9^	ND in AA
KW-0007~Acetylation	7.1 × 10^−5^	Septin-4 (SEPTIN4)	5.2 × 10^−10^	ND in AA
GO:0005739~mitochondrion	7.2 × 10^−4^	Sperm equatorial segment protein 1 (SPESP1)	2.4 × 10^−6^	0.003
GO:0031514~motile cilium	9.3 × 10^−3^	Ubiquitin carboxyl-terminal hydrolase 5 (USP5)	1.9 × 10^−3^	0.4
KW-0597~Phosphoprotein	2.2 × 10^−3^	Annexin A1(ANXA1)	2.3 × 10^−3^	0.03
GO:0005856~cytoskeleton	4.5 × 10^−3^	Peroxiredoxin-1 (PRDX1)	2.5 × 10^−3^	0.7

The table summarizes the highly enriched biological functions with selected significant differentially expressed proteins in the young adult (21 in Cluster 2 and 15 in Cluster 3), late adult (36 in Cluster 4 and 32 in Cluster 9), and advanced age (14 in Cluster 6 and 45 in Cluster 8) groups. YA: young adult (21–30 years); LA: late adult (31–40 years); AA: advanced age (41–51 years); *p*-values: derived from ANOVA; FDR: false discovery rate; ND: not detected; YA/AA, LA/AA, and AA/LA represent fold ratios.

## Data Availability

Data is contained within the article and Supplementary Materials. The mass spectrometry proteomics data have been deposited to the ProteomeXchange Consortium via the PRIDE partner repository with the dataset identifier PXD062650 and 10.6019/PXD062650.

## Supplementary Materials

The following supporting information can be downloaded at: https://www.mdpi.com/article/10.3390/ijms26136099/s1.
